# Identification of Novel Causal FBN1 Mutations in Pedigrees of Marfan Syndrome

**DOI:** 10.1155/2018/1246516

**Published:** 2018-04-17

**Authors:** Yueli Wang, Xiaoyan Li, Rongjuan Li, Ya Yang, Jie Du

**Affiliations:** Department of Echocardiography, Beijing Anzhen Hospital, Capital Medical University and Beijing Institute of Heart, Lung and Blood Vessel Diseases, Beijing, China

## Abstract

Marfan syndrome (MFS) is an autosomal dominant genetic disorder of the connective tissue, typically characteristic of cardiovascular manifestations, valve prolapse, left ventricle enlargement, and cardiac failure. Fibrillin-1 *(FBN1*) is the causative gene in the pathogenesis of MFS. Patients with different *FBN1* mutations often present more considerable phenotypic variation. In the present study, three affected MFS pedigrees were collected for genetic analysis. Using next-generation sequencing (NGS) technologies, 3 novel frameshift pathogenic mutations which are cosegregated with affected subjects in 3 pedigrees were identified. These novel mutations provide important diagnostic and therapeutic insights for precision medicine in MFS, especially regarding the lethal cardiovascular events.

## 1. Introduction

Marfan syndrome (MFS; OMIM 154700) is a common autosomal dominant connective tissue disorder; the incidence rate was estimated to be at least 1 per 10,000 individuals without any racial, geographical, or occupational predilection. This disorder affects cardiovascular, ocular, and skeletal systems, as well as skin, lung, and central nervous systems. Mortality and morbidity are mainly determined by the development of cardiovascular events, such as heart failure, aortic aneurysm, and subsequent aortic dissection [1].

MFS was caused by fibrillin-1 (FBN1) gene mutations (NM_000138), which is located on chromosome 15q21.1 and had 65 exons. Fibrillin-1, a kind of extracellular matrix glycoprotein, was reported as an important calcium-binding microfibrillar structural molecule and a regulator of TGF-*β* signaling [2]. It consists of 47 epidermal growth factor-like (EGF) domains, 7 transforming growth factor *β*-1-binding protein-like (TB) domains, and a heterozygous domain. To date, about 3000 mutations [3] have been detected in *FBN1* and mainly classified into 3 types including missense mutations, in-frame deletions, and nonsense mutations due to frameshift leading to premature termination codons (PTC) [4, 5], even though progresses had been made for the detection of causal mutations in MFS.

Studies have demonstrated a higher probability of cardiovascular events in patients with mutations reducing the amount of *FBN1* (classified as HI mutation). These genotypes cause severity and worse prognosis, including increased risk for aortic surgery, aortic dissection, and mortality. The genotype–phenotype effect was recognized as an important factor when making a diagnosis of MFS and later predictable phenotype severity as well as clinical decisions [6, 7]. Some have argued that truncating may be associated with a milder disease course, which has long been questioned [8, 9]. For precision medicine of prognosis and treatment of MFS patients, more studies on the genotype–phenotype effect are warranted.

Here, 3 pedigrees with MFS were selected to identify novel mutations and assess genotype–cardiovascular phenotype effects. 3 novel, not yet reported, pathogenic frameshift mutations in *FBN1* were identified, which may contribute to providing diagnostic values in precision medicine of the genotype–cardiovascular phenotype relationship.

## 2. Material and Methods

### 2.1. Patients and Clinical Data

**Figure 1:**
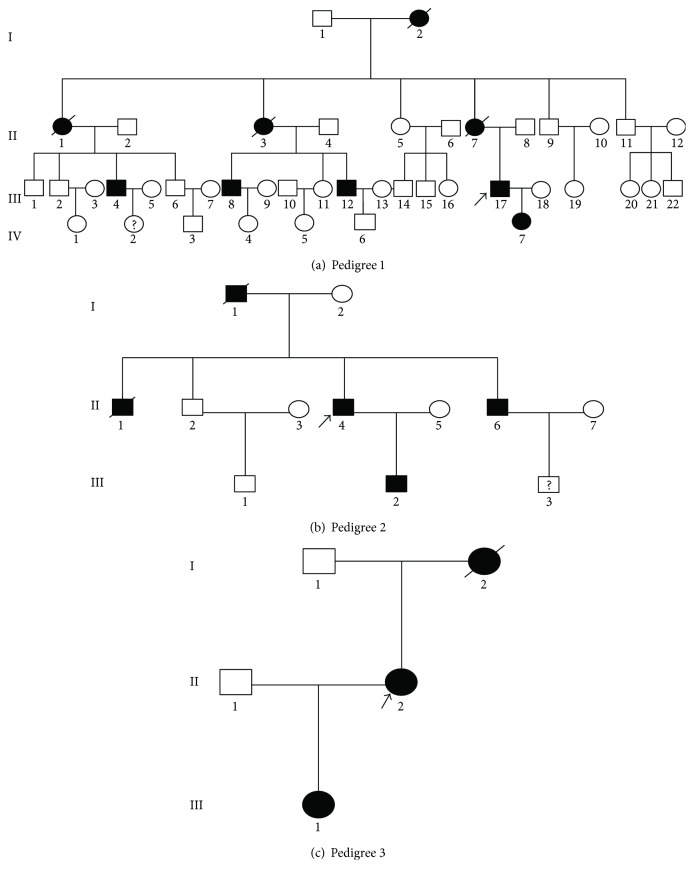
The proband pedigrees with MFS. +/− represents the heterozygous type; −/− represents the wild type. “Male” and “female” are indicated by squares and circles, respectively, and the filling symbols represent individuals affected with Marfan syndrome. The arrow shows the proband.

In this study, 3 ethnic Han Chinese families were recruited in Beijing Anzhen Hospital and diagnosed as MFS according to the revised Ghent nosology [10] based on their reported family history, clinical features, and essential echocardiography examinations. The study was approved by the ethics committee of Beijing Anzhen Hospital and performed according to the tenets of the Declaration of Helsinki. 3 pedigrees are shown in Figure 1. Detailed clinical information of all patients is listed in Table 1.

### 2.2. Genetic Testing Panel for Marfan Syndrome and Next-Generation Sequencing

A specific target sequencing panel was designed for the maximum coverage mutation of MFS. The target genes include FBN1, TGFBR1, and TGFBR2. Probes were designed and synthesized according to the Roche NimbleGen SeqCap EZ Choice manuscript. Genomic DNA was nebulized before adaptor ligation. Size selection was carried out using AMPure XP beads with a target size of 200 to 300 bp. Whole-genome shotgun libraries were prepared using the Illumina TruSeq Paired-End Prep Kit (Illumina Inc., San Diego, CA, USA) according to the manufacturer's protocol. The three DNA libraries were pooled together and hybridized to NimbleGen SeqCap EZ panel to enrich the target region.

Samples were sequenced on HiSeq 2500 (Illumina) following the manufacturer's recommendations, in paired-end mode for 100 cycles.

### 2.3. Sanger Resequencing

The mutations detected with NGS were confirmed by Sanger sequencing using the BigDye Terminator v3.1 Cycle Sequencing kit (Applied Biosystems; Thermo Fisher Scientific Inc.), followed by capillary electrophoresis on an 3500xL Genetic Analyzer (Applied Biosystems; Thermo Fisher Scientific Inc.). The identified mutations were further targeted for Sanger sequencing (cascade testing) in other family members from these 3 pedigrees, respectively.

### 2.4. Function Prediction of Gene Mutation Function

A forecasting software, MutationTaster, was used to predict the effect of the functional importance of variation loci identified in 3 pedigrees. MutationTaster provides a probability for the alteration to be either a causative mutation or a benign polymorphism [11].

## 3. Results

### 3.1. Clinical Findings of the Pedigree

#### 3.1.1. Concerning Family 1

**Figure 2:**
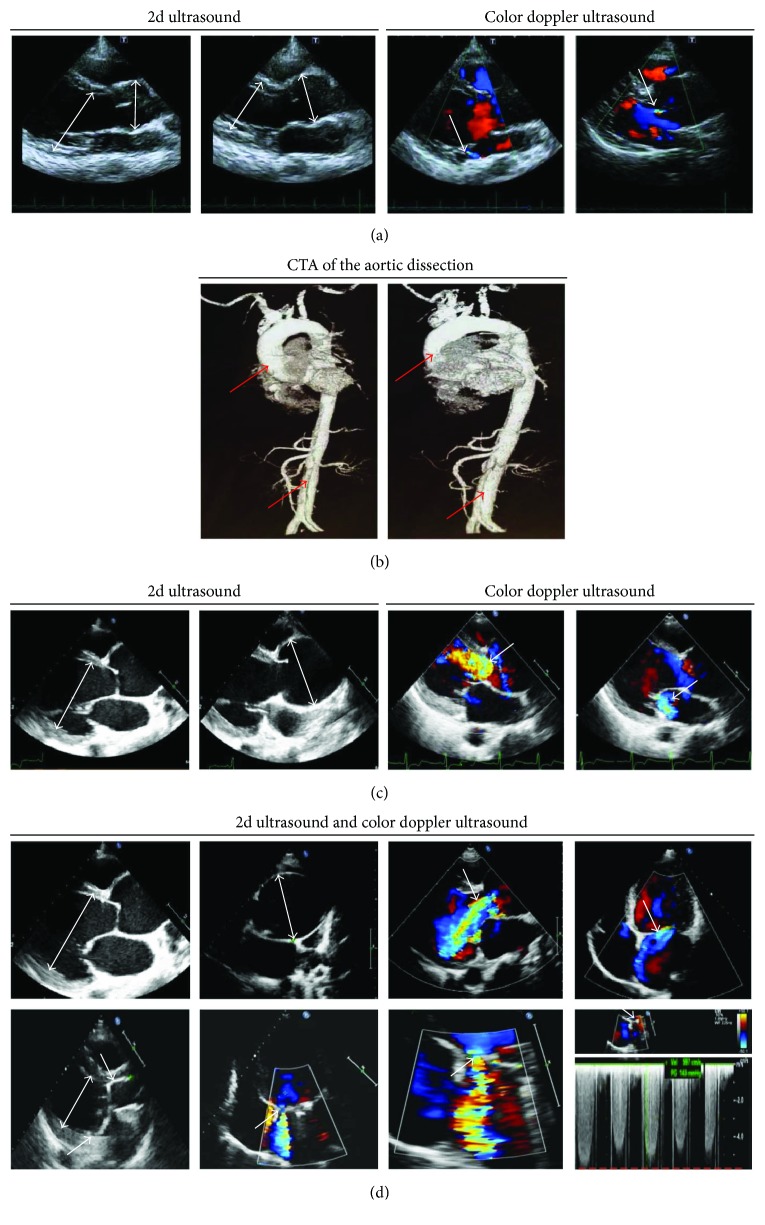
Echocardiography and computed tomography angiography findings for patients. (a) Transthoracic echocardiography at first diagnosis, showing a slight aortic root dilatation (arrow), mild aortic regurgitation (AR, arrow), and mild mitral regurgitation (MR, arrow) of proband in pedigree 1. (b) Aortography results after first checkup at 2 months. Transthoracic echocardiography at first diagnosis of proband in pedigree 2, showing a remarkable aortic root dilatation (arrow), massive aortic regurgitation, and mild mitral regurgitation (arrow). (d) Preoperative (top) and postoperative (bottom) transthoracic echocardiography of individual II: 6 in pedigree 2. Preoperative echocardiographic characteristics (top) showed a remarkable aortic root dilatation (arrow), massive aortic regurgitation, and mitral regurgitation (arrow). Transthoracic echocardiography after the first cardiac surgery (bottom) showed a moderate mitral periprosthetic leak (arrow) and still enlarged left ventricle despite a replaced artificial double valve (DVR).

We analyzed a four-generation Marfan syndrome family composed of 9 affected members including 4 males and 5 females. Target gene sequencing was used to detect the proband and his 8-year-old daughter, both manifesting a marfanoid aortic sinus and tall stature. The proband's wife (27-year-old), an unaffected family member, had no clinical features of MFS. Clinical data of the patients are shown in Table 1. The proband (29-year-old), with a 187 cm height and all lanky as well as leggy body, presented to our hospital due to chest tightness and shortness of breath. Transthoracic echocardiography (Figure 2(a)) revealed aortic root dilatation (aortic diameter at the sinuses of Valsalva: 45 mm), mild aortic valve regurgitation, and normal left ventricular end diastolic diameter (LVED). These recommendations do not meet the formal criteria for prophylactic aortic surgery, so conservative therapy is suggested [12]. However, unexpected type A acute aortic dissection developed 2 months later (Figure 2(b)). The proband's mother was described to have suffered from sudden death at the age of 35. As for other relations, individuals I: 1, II: 1, II: 3, and II: 7 passed away. Individuals III: 4, III: 8, and III: 12 suffered from aortic aneurysm/dissection; clinical data are shown in Supplementary Table 1. Therefore, we speculated that inherited mutation in the family is responsible for a young, rapid-progressing, and extremely dangerous aortic dissection phenotype.

#### 3.1.2. Concerning Family 2

6 of 12 members in this 3-generation family were diagnosed as having MFS. Clinical data of the patients are shown in Table 1. The proband, with an average height, complained of paroxysmal chest pain and heart murmurs. TTE evaluation (Figure 2(c)) presented severe aortic root dilatation (aortic diameter at the sinuses of Valsalva 60 mm), severe aortic valve regurgitation, moderate mitral insufficiency, enlarged LVED, and poor left ventricular ejection fraction (LVEF: 35%), indicating that prophylactic aortic surgery was urgently needed according to the 2014 ESC Guidelines [12]. Since the dilatation of the aortic root was causing aggravation and the aortic valve developed severe regurgitation, the patient underwent a surgical operation for aortic valve and mitral valve replacement. The proband's 14-year-old son (Individual III: 2) (Table 1) not only displayed similar cardiovascular indicators (including marfanoid aortic sinus, enlarged LVED, and moderate mitral valve prolapse as well as mild tricuspid valve prolapse) as his father, but also was 185 cm tall and highly myopic, just like family 1 presented.

The young brother (individual II: 6) of the proband exhibited similar clinical and echocardiographic features (Table 1 and Figure 2(d)), dilatation of the aortic sinus to 67 mm with marked aortic valve regurgitation, and moderate mitral insufficiency. Individual II: 6 underwent the same surgery as the proband, but with a poor prognosis (Figure 2(d)). Individuals I: 1 and II: 1 died due to cardiac events. Taken together, family 2 have different features on cardiovascular disorders rather than early aortic dissection.

#### 3.1.3. Concerning Family 3

The proband (individual II: 2) was a 29-year-old woman who presented typical marfanoid cardiovascular features, facial features, and arachnodactyly, but without ectopia lentis. Echocardiography detected a dilative aortic sinus, with a diameter of 43 mm. Her 5-year-old daughter (individual III: 1) complained of pectus excavatum and was the first to be diagnosed with MFS in Beijing Children's Hospital 2 years before. The mother of the proband had a sudden death at the age of 45.

Further comparing the similarities and differences among these 3 families showed that there is no evidence of skeletal morbidity (including arm span to height > 1.05, scoliosis, and joint hypermobility) in either of the 3 families. Therefore, it is possible to propose that MFS is variable both among and within affected families in severity of cardiovascular manifestations, due to type of mutation.

### 3.2. Mutation Identification and Bioinformatic Analysis

**Figure 3:**
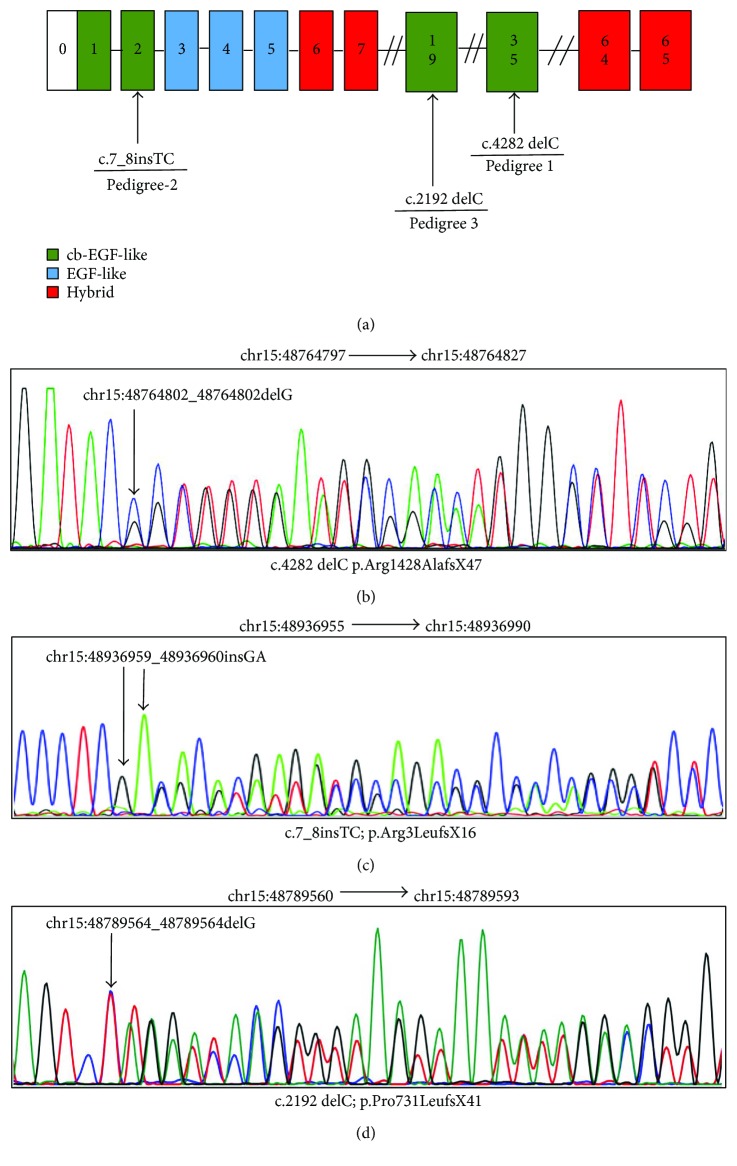
Localization of mutations in FBN1 and results from qualitative analysis. (a) Schematic presentation of FBN1 with the localization of the 3 mutations investigated in this study indicated. (b) Fragment of FBN1 cDNA sequence in a patient with c.4282 delC. (c) Fragment of FBN1 cDNA sequence in a patient with c.7_8insTC. (d) Fragment of FBN1 cDNA sequence in a patient with c.2192 delC.

In 3 families diagnosed with MFS, we identified independent heterozygous frameshift mutations of FBN1 (Figure 3(a)). None of the mutations existed in known databases (UMD-FBN1, HGMD, ClinVar, UCSC common SNP, db SNP, and the 1000-genome project) or in published articles.

**Figure 4:**
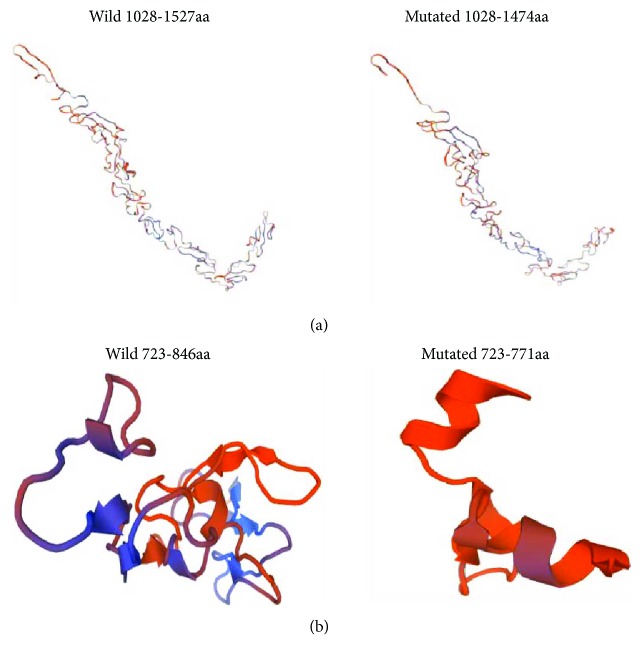
Predicted results for the frameshift variants by Swiss-Model. (a) Predicted 3D model of the variant (c.4282 delC.) in family 1. (b) Predicted 3D model of the variant (c.2192 delC.) in family 3.

A novel frameshift deletion c.4282delC (p.Arg1428AlafsX47) in CDS34 (chr15:48764802) was identified in family 1 individuals III: 17 and IV: 7 (Figure 3(b)). According to Sakai et al. reported in 2006, a mutation c.4283dup (p.Cys1429LeufsX2) was observed in a Marfan patient from Japan [13]. Further analysis indicated that this mutation leads to truncation of the original 2871-amino acid full-length protein to a 1474-residue protein. This mutation is located between the epidermal growth factor- (EGF-) like domain, according to UniProtKB [14]. We used two algorithmic tools, MutationTaster and Swiss-Model, to examine the impact of this mutation. In the conservation test by MutationTaster [15], the affected residue in family 1 was revealed to be evolutionarily conserved, which implied that the alternation of amino acids in this position is likely to damage protein function. Furthermore, the mutant tertiary structure generated by Swiss-Model predicts that the domain where the mutation is located (1028–1474) differs from that of the wild-type protein (1028–1527) (Figure 4(a)). Therefore, this frameshift mutation was identified as being rare, probably disease causing, which might affect protein function by influencing the structure of the FBN1 protein or the binding between calcium and the cbEGF domain.

In family 2 individuals II: 3 and IV: 2, one de novo frameshift mutation, c.7_8insTC (p.Arg3LeufsX16), in exon 2 of FBN1 was identified (Figure 3(c)). This event resulted in the insertion of 2 base pairs, leading to a change from amino acids 3 to 18 and a deletion of big fragments from amino acids 19 to 2871, which could likely damage protein function overwhelmingly. Furthermore, MutationTaster showed this mutation to be pathogenic.

The sequencing results from family 3 individual II: 2 revealed a heterozygous frameshift mutation c.2192 delC (p.Pro731LeufsX41) in CDS18 of FBN1 (Figure 3(d)), which caused a translation of the protein to stop at the 771st amino acid residue, by the premature termination codon (PTC). Refer to UniProtKB; this mutation is also located between the cb EGF-like domain. The disease-causing potential of this variation was predicted automatically by MutationTaster. Swiss-Model predicts that the domain where the variant is located (723–771) is significantly different from that of the wild-type protein (723–846) (Figure 4(b)). These results indicate that c.2192 delC led to a truncation of the protein, which is associated with cardiovascular phenotype thoracic aortic aneurysms and dissections.

## 4. Discussion

Most patients with MFS will ultimately develop cardiovascular outcomes (aortic aneurysm, valve prolapse as well as LV dysfunction, etc.) and might further progress into aortic dissection, owing to the clinical variability in FBN1 gene mutations. Thereby, uncovering novel causal mutations and genotype–cardiovascular phenotype correlations would facilitate genetic counselling and allow early symptomatic treatment, in order to improve the prognosis of patients eventually [16].

In the present study, we selected 3 families with MFS and identified novel FBN1 mutations. Intriguingly, 3 novel frameshift PTC mutations caused typical and variable cardiovascular complications in affected family members, from severe to mild.

Several genotype–phenotype correlations in cardiovascular involvement have been established. The early observation by Aoyama et al. showed that mutations leading to a very low deposition of the fibrillin-1 protein were associated with shortened event-free survival and more severe cardiovascular complications [17]. In addition, some previous publications have demonstrated a strong association of MFS patients with truncating FBN1 mutations with cardiovascular events [6, 18]. Moreover, recent reports discovered that patients with premature termination codon (classifiable as HI) have a worse prognosis, with increased risk for aortic surgery, aortic complications, and mortality compared with DN mutation carriers [19–21]. Alternatively, Wang et al. likewise observed an association with truncating mutations in MFs with cardiovascular defects [22]. For a severe ventricular phenotype, like LV dilatation, it has been reported to be related with the disorder of microfibril assembly by in-frame deletions or non-missense mutations [23, 24]. Likewise, the present study also identified similar results as in previous studies in 3 MFS pedigrees with frameshift mutations, thereby contributing to the extension of the known mutational spectrum of frameshift, for understanding the genotype–phenotype correlations in cardiovascular involvement.

In the current study, 3 novel frameshift variants, c.4282delC (p.Arg1428AlafsX47), c.7_8insTC (p.Arg3LeufsX16), and c.2192 delC (p.Pro731LeufsX41), appear to have a significant association with cardiovascular involvement and mild skeletal manifestations. Additionally, evidence from 3 pedigrees also showed varying degrees of cardiovascular phenotype owing to their own unique HI mutation. The mutation c.4282delC (p.Arg1428AlafsX47) in family 1 affects protein function by the destroyed protein structure and functions for binding of calcium and causes severe manifestations involving rapid but early onsets of aortic dissection, minor findings in both ocular and skeletal systems. Thus, c.4282delC might serve as a potential target for future research on patients with rapidly progressive aortic dissection. Another case of family 2, the proband and his young brother, though with a predominant dilated aortic sinus and LV enlargement (latent LV dysfunction, valvular insufficiency, and subsequent worse prognosis after surgery (Figure 4), have a survival time free of dissection until now. Interestingly, it is known that another mutation located on exon 2 in FBN1 results in a frameshift and premature termination of translation, and the proband likewise had major findings in the cardiovascular system, involvement of the skeletal system, and minor findings in the ocular system [21]. c.2192 delC (p.Pro731LeufsX41) mutation in patient II: 2 from family 3, which leads to a cb EGF-like domain destruction as well, just displayed a typical marfanoid dilation of aortic sinus at present, which should be monitored, since both the proband and her daughter are young, and the clinical phenotypes are not only variable but time-dependent.

Altogether, this study added 3 novel mutations to the existing spectrum of FBN1 mutations and demonstrated different frameshift genotype impacts on cardiovascular-phenotype severity in patients with MFS, which might facilitate understanding on potential phenotype–genotype associations and refine susceptible populations of deleterious cardiovascular events such as greater risk for early aortic dissection or markedly poor heart function even after prophylactic surgery. Therefore, extensive clinical and basic researches on the FBN1 mutation effects on fibrillin-1 protein are still warranted for precision medicine of cardiovascular risk classification, preventing higher-risk adverse outcomes and also avoiding unnecessary interventions in diverse patients with MFS, so as to achieve an increasingly tailored patient-specific management eventually.

## Figures and Tables

**Table 1:** Clinical detail of the patients with FBN1 mutation in this study.

Individuals	Pedigree 1		Pedigree 2			Pedigree 3	
	III: 17	IV: 7	II: 6	II: 6	III: 2	II: 2	III: 1
Age (years)	33	10	45	41	14	29	5
Gender	Male	Female	Male	Male	Male	Female	Female
Height (cm)	187	151	173	175	185	182	130
Ocular system							
Ectopia lentis	−	−	+	−	−	−	−
Myopia	+	+	−	−	+	−	−
Cardiovascular system		−					
Diameter of aortic root (mm)	45	30	60	67	36	43	^∗^
Aortic dissection	+	−	−	−	−	−	^∗^
Mitral valve prolapse	−	−	−	+	+	−	^∗^
Tricuspid valve prolapse	−	−	−	−	+	−	^∗^
LVED (mm)	53	41	72	81	58	49	^∗^
LVEF (%)	60	62	50	35	54	66	^∗^
Systemic features							
Thumb sign	+	+	−	−	+	+	^∗^
Wrist sign	+	+	−	−	+	+	^∗^
Thin body	+	+	+	+	+	+	^∗^
Arachnodactyly	+	+	−	−	+	+	^∗^
Pectus excavatum; scoliosis	−	−	−	−	−	−	+

∗ represents unknown information.

## Supplementary Materials

Supplementary Materials Table 1: clinical data of other 3 patients (individuals III: 3, III: 8, and III: 12) in the first pedigree.
