# Supplementary material for: Identification of Novel Causal FBN1 Mutations in Pedigrees of Marfan Syndrome
Source: Int J Genomics. 2018 Apr 17;2018:1246516. doi: 10.1155/2018/1246516 (PMC5932419; doi:10.1155/2018/1246516)
Supplement: Supplementary Materials — Table 1: clinical data of other 3 patients (individuals III: 3, III: 8, and III: 12) in the first pedigree. [file 1246516.f1.doc]

**Table S1** Clinical data of other 3 patients(Individuals III:3, III:8, III:12) in the first pedigree.





The "*" sign represents the unknown information.
